# Critical role of neutralizing antibody for SARS-CoV-2 reinfection and transmission

**DOI:** 10.1080/22221751.2021.1872352

**Published:** 2021-01-19

**Authors:** Young-Il Kim, Se-Mi Kim, Su-Jin Park, Eun-Ha Kim, Kwang-Min Yu, Jae-Hyung Chang, Eun Ji Kim, Mark Anthony B. Casel, Rare Rollon, Seung-Gyu Jang, Jihye Um, Min-Suk Song, Hye Won Jeong, Eung-Gook Kim, Yeonjae Kim, So Yeon Kim, Jun-Sun Park, Mi Sun Park, Geun-Yong Kwon, Sang Gu Yeo, Shin-Ae Lee, Youn Jung Choi, Jae U. Jung, Young Ki Choi

**Affiliations:** a College of Medicine and Medical Research Institute, Chungbuk National University, Cheongju, Republic of Korea; b Zoonotic Infectious Diseases Research Center, Chungbuk National University, Cheongju, Korea; c Division of Life Science and Research Institute of Life Sciences, Gyeongsang National University, Jinju, Korea; d Research institute of Public Health, National Medical Center, Seoul, Republic of Korea; e Div. of Public Health Research, Sejong Institute of Health & Environment, Sejong City, Republic of Korea; f Sejong Public Health Center, Sejong City, Republic of Korea; g Department of Cancer Biology and Center for Global and Emerging Pathogen Research, Lerner Research Institute, Cleveland, OH, USA

**Keywords:** SARS-CoV-2, reinfection, COVID-19, neutralizing antibody, ferret model

## Abstract

Cases of laboratory-confirmed SARS-CoV-2 reinfection have been reported in a number of countries. Further, the level of natural immunity induced by SARS-CoV-2 infection is not fully clear, nor is it clear if a primary infection is protective against reinfection. To investigate the potential association between serum antibody titres and reinfection of SARS-CoV-2, ferrets with different levels of NAb titres after primary SARS-CoV-2 infection were subjected to reinfection with a heterologous SARS-CoV-2 strain. All heterologous SARS-CoV-2 reinfected ferrets showed active virus replication in the upper respiratory and gastro-intestinal tracts. However, the high NAb titre group showed attenuated viral replication and rapid viral clearance. In addition, direct-contact transmission was observed only from reinfected ferrets with low NAb titres (<20), and not from other groups. Further, lung histopathology demonstrated the presence of limited inflammatory regions in the high NAb titre groups compared with control and low NAb groups. This study demonstrates a close correlation between a low NAb titre and SARS-CoV-2 reinfection in a recovered ferret reinfection model.

## Introduction

In December 2019, dozens of patients with pneumonia were reported in Wuhan, China [1]. On January 8, 2020, the infectious agent was identified as a novel coronavirus (2019-nCoV), which was named severe acute respiratory syndrome (SARS) coronavirus-2 (SARS-CoV-2) due to its marked similarity, in terms of clinical symptoms and biological nature, to the causative agent of severe acute respiratory syndrome coronavirus (SARS-CoV) first reported in 2002 [1,2]. The World Health Organization (WHO) declared SARS-CoV-2 a pandemic on March 11, 2020, as the exponential increase of SARS-CoV-2 infection cases in Asia, Europe, and North America posed a serious threat worldwide [3]. As of December 6, 2020, the global total confirmed cases are 65,870,030 with 1,523,583 recorded deaths, and cases are still on the rise [4].

Despite numerous ongoing clinical trials to evaluate vaccine candidates and to repurpose drugs for the prevention and treatment of SARS-CoV-2 infection, there are not clear treatment options and vaccination at levels required for herd immunity will take considerable time. In order to keep this pandemic under control in the absence of licensed vaccines and therapeutics, some have proposed attainment of SARS-CoV-2 herd immunity through natural infection [5]. However, there is currently no data that shows patients who have recovered from SARS-CoV-2 infection are protected from re-exposure [6]. Furthermore, even if a protective immune response is developed, the duration of protective immunity against SARS-CoV-2 infection is unknown [7]. Recently, Wu et al. reported that out of 175 recovered COVID-19 patients, about 30% failed to develop high neutralizing antibody titres, and 10 patients showed very low or undetectable levels of neutralizing antibodies [8]. Longitudinal studies on Middle East Respiratory Syndrome-Coronavirus (MERS-CoV) have also indicated that serum antibody titres wane over time, particularly following mild infections [9]. Similar trends were also observed in classic SARS-CoV infections [10,11]. Although a recent study in rhesus macaques did not find evidence of reinfection from subsequent exposure after recovery from SARS-CoV-2 infection [12], human coronavirus NL63 (HCoV-NL63) exhibited reinfection potentials without genotype switching, where in some cases, the second infection yielded a higher viral load [13]. Thus, it appears that initial exposure to HCoV-NL63 may not elicit sufficient protective immune responses. Moreover, Houser et al. reported that in a rabbit model, antibodies against MERS-CoV proteins lack neutralizing activity, resulting in reinfection with enhanced pulmonary inflammation [14]. This is similar to Dengue virus infection and other coronavirus infections such as feline infectious peritonitis [15,16].

Although cases of suspected SARS-CoV-2 reinfection have continuously been rising among recovered COVID-19 patients [17,18], their immune responses against the virus, especially the role of serum neutralizing antibody (NAb), have not been well characterized. In this study, to determine the correlation between NAb titres and reinfection rate, we adapted a ferret reinfection model with dose-dependent SARS-CoV-2 NAb to evaluate virus replication, shedding periods, and changes in antibody titres during the heterologous SARS-CoV-2 reinfection period. This study reveals that NAb titre is a critical factor for SARS-CoV-2 reinfection in the ferret model.

## Materials and methods

### Isolation of infectious virus from specimens

Specimens collected from SARS-CoV-2-infected ferrets were used to infect Vero cells (ATCC, CCL-81) for virus isolation. Briefly, specimens were centrifuged at 4°C at 1200 rpm for 15 min and the supernatants were incubated with Vero cells for 2 h. Media (DMEM) was changed daily and cells were monitored for 4 days to examine the cytopathic effects (CPEs). To confirm virus isolation, we performed qRT-PCR on supernatants from infected cell cultures using S gene-specific primer sets [Forward (5′-3′): AGGGCAAACTGGAAAGATTGCTGA, Reverse (5′-3′): GTTCTTTATCAGGATGTTAACTGCACAGA; 569 bp]. All RT-PCR positive specimens were confirmed by sequencing.

### Ferret infection and grouping by serum neutralization antibody (NAb) titre

**Figure 1.**
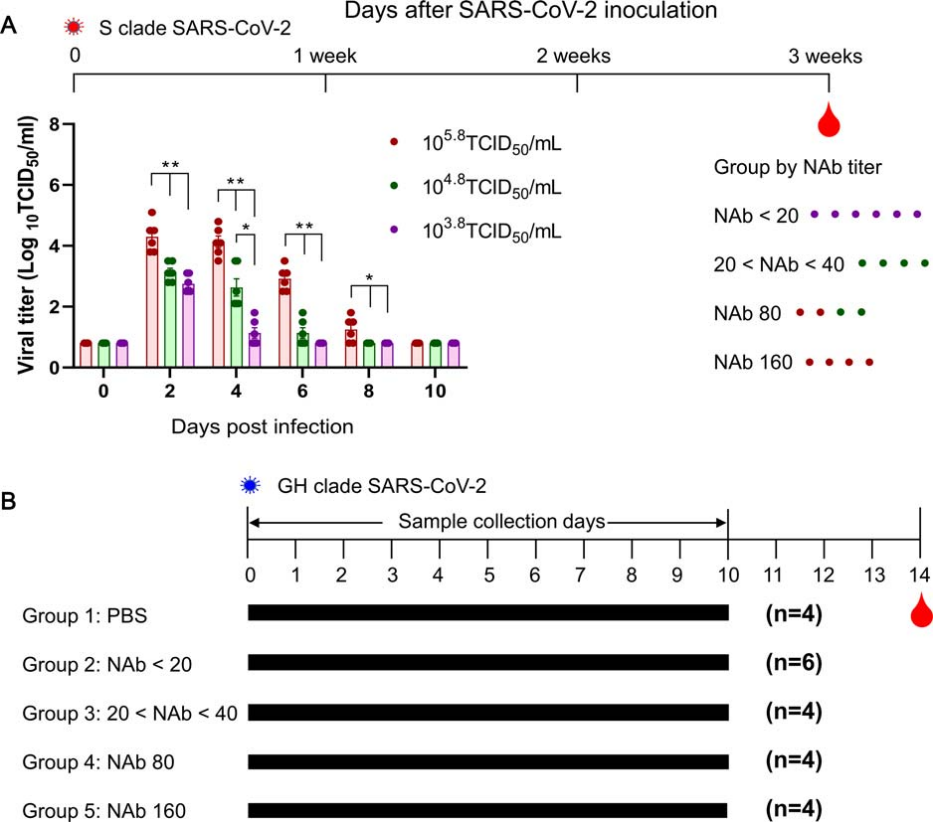
Schedule of SARS-CoV-2 pre- and reinfection in ferrets. To induce varied immune responses, three different doses of SARS-CoV-2 were used to infect groups of ferrets. Following three weeks of infection, serum NAb titres were measured in Vero cells, and ferrets were then grouped according to their NAb titres (NAb < 20, 20–40, 80, and 160) (A). Asterisks indicate statistical significance between each infection group as determined by two-way ANOVA Tukey’s multiple comparisons test (* indicates *p* < 0.05, ** indicates *p* < 0.0001). Each group of NMC-nCoV02 (S clade)-primed ferrets and the naive control group were inoculated intranasally with 10^5.0^ TCID_50_ of CBNU-nCoV02 (GH clade) followed by virus and blood collection on day 14 post-infection (B).

Ferrets, 12 to 24-months-old and confirmed negative for influenza A viruses (H1N1, H2N2), MERS-CoV and SARS-CoV antibodies, were used for primary infection. Briefly, three different groups of ferrets (*n* = 6) were inoculated through the intranasal route with NMC-2019-nCoV02 virus, at doses of 10^5.8^, 10^4.8^, and 10^3.8^ TCID_50_/mL for each ferret group. Three weeks after virus infection, a serum NAb assay was conducted as described below. Ferrets were then grouped according to their NAb titres (NAb < 20 (G2), 20–40 (G3), 80 (G4), 160 (G4)) including a naive control group (G1) (Figure 1A).

### Reinfection of immunized ferrets with heterologous SARS-CoV-2 strain

Each group of ferrets was inoculated through the intranasal route with a CBNU-nCoV02 virus, a heterologous virus with 99.9% homology (Table 1) with NMC-2019-nCoV02 in the spike protein, at a dosage of 10^5.0^ TCID_50_/mL for ferret. As a control group, three naïve ferrets were also infected with CBNU-nCoV02. Nasal washes and rectal swabs were collected from anesthetized ferrets at 2-day intervals until 10 days post-infection (dpi) to determine the viral load by qRT-PCR. For virus isolation, collected specimens were inoculated with Vero cells as described above.

**Table 1.** Comparison of SARS-CoV-2 gene mutations of the S and GH clade human isolate.

SARS-CoV-2 (Ref. WIV04)	ORF1a		ORF1b		S		ORF3		ORF8		Homology of amino acids
	n.t	a.a	n.t	a.a	n.t	a.a	n.t	a.a	n.t	a.a	
NMC-nCoV02 (S clade)	T2821C C5990T C8517T G10818T	F941L A1987V –* L3602F	G3681A	A293T					T251C	L84S	99.9%
CBNU-nCoV02 (GH clade)	C2772T	–	C967TC5436T	– –	C733T A1841G	H243Y D614G	G171T	Q57H			

Notes: Nucleotide or amino acid substation are presented based on the first SARS-CoV-2 isolate (hCoV-19/Wuhan/WIV04/2019) as a reference strain (L clade).

*Synonymous mutation.

### Histology

Lung tissue samples were collected for each group at 6 dpi and were incubated in 10% neutral-buffered formalin for fixation before they were embedded in paraffin based on standard procedures. Tissue sections were then placed on glass slides and stained with hematoxylin and eosin (H&E). Slides were viewed using an Olympus IX 71 (Olympus, Tokyo, Japan) microscope with DP controller software to capture images.

### Serum neutralizing antibody (NAb) assay for SARS-CoV-2

To evaluate the neutralization titre of the collected specimens, a serum NAb assay against SARS-CoV-2 (Korean isolates; NMC-nCoV02 and CBNU-nCoV02) was performed in a BSL3 facility. Heat-inactivated ferret serum samples were serially diluted by two-fold. An equal volume of SARS-CoV-2 at 100 TCID_50_ was incubated with all diluted samples for 1 h at 37 °C followed by inoculation in Vero cells. After 1 h of inoculation, the serum and virus mixture was removed and DMEM was added to the infected cells. The cells were incubated at 37°C in 5% CO_2_ and monitored for CPE. After 4 days, supernatants were removed and cells were fixed with 10% formalin solution, followed by staining with crystal violet to determine the titre. Antibody titres were defined as the highest serum dilution that inhibited CPE. A 1:10 dilution was considered as the lowest possible significant titre.

### Indirect immunofluorescence analysis (IFA)

Vero cells were infected with 1 × 10^2^ TCID_50_ of NMC-2019-nCoV02 and CBNU-nCoV02 for 2 h at 37°C and incubated for 2 days. The infected cells were fixed with 4% formaldehyde prior to permeabilization with 1% Triton X-100 (Sigma, St. Louis, USA) in PBS and blocking with 3% BSA in PBS. After washing five times, diluted serum (1:10) samples were incubated with fixed cells for 3 h at 37°C, and IgG detected using a fluorescein-labeled antibody against ferret IgG (Abcam, Cambridge, England). Fluorescence was observed using an Olympus IX 71 (Olympus, Tokyo, Japan) microscope and DP controller software to capture images.

### Ethics statement

For animal studies, male and female ferrets were maintained in isolators in the BSL3 laboratory. All animal studies were carried out in accordance with protocols approved by the Institutional Animal Care and Use Committee (IACUC) at Chungbuk National University (Approval number CBNUA-1352-20-02).

## Results

### Demonstration of SARS-CoV-2 reinfection in ferrets

It remains unknow if antibody production in recovered SARS-CoV-2 infected patients can afford protection against re-exposure to heterologous SARS-CoV-2 strains and, if reinfection does occur, if SARS-CoV-2 reinfected patients shed infectious virus. To answer these questions, we adapted the ferret model, which is highly susceptible to SARS-CoV-2 infection and transmission [19,20] and evaluated virus titres, shedding periods, and changes in antibody titres prior and subsequent to reinfection.

For pre-infection, groups of ferrets (*n* = 6) were infected with NMC-2019-nCoV02 virus (S clade) at doses of 10^5.8^, 10^4.8^, or 10^3.8^ TCID_50_/mL for each ferret. To confirm the virus infection, nasal washes were collected at 2, 4, 6, 8, and 10 days post-infection (dpi) for virus titration. The 10^5.8^ TCID_50_/mL infection group revealed the highest virus titres, which persisted until 8 dpi while the 10^3.8^ TCID_50_/mL infection group showed the lowest virus titres, which only persisted until 4 dpi (Figure 1A). Three weeks after virus infection, serum neutralizing antibody (NAb) titres were measured in Vero cells. All ferrets infected with the 10^5.8^ TCID_50_/mL dose showed NAb titres as high as 80–160, while those infected with 10^3.8^ TCID_50_/mL showed NAb titres less than 20.

**Figure 2.**
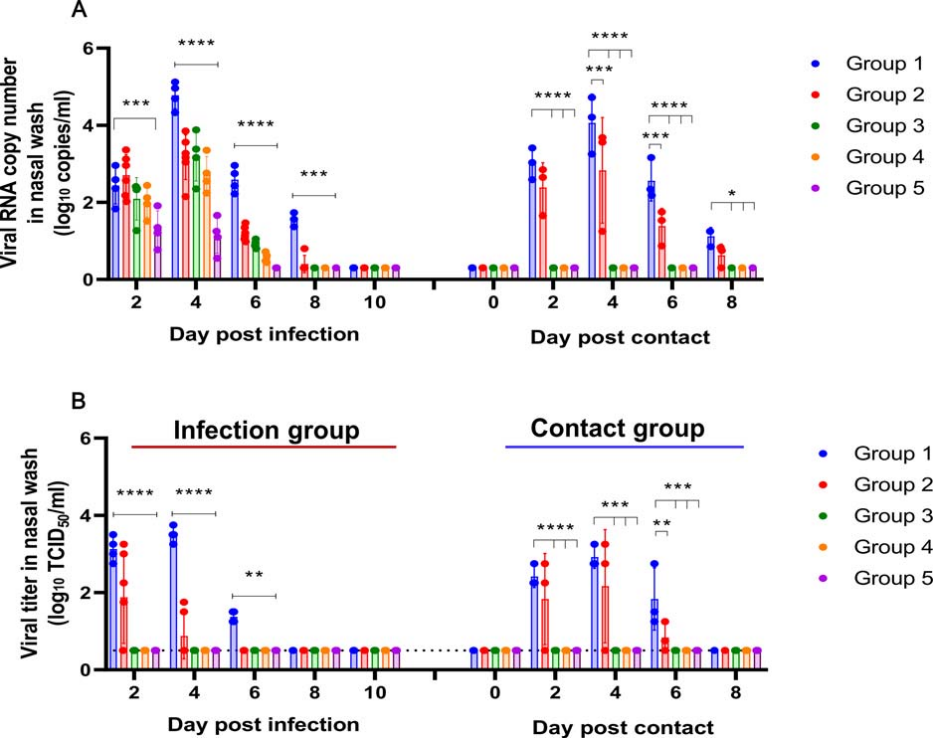
Nasal wash virus titres of ferrets. Virus titres were measured in nasal washes of CBNU-nCoV02-infected ferrets and direct contact sentinel ferrets at 2, 4, 6, 8, 10 days of reinfection. Viral loads in nasal washes were measured by TCID_50_ (A). The virus RNA copy numbers were measured with qRT-PCR (B). The limit of viral RNA detection with qRT-PCR is 0.3 log_10_ copies/mL copies per reaction. Data is presented as mean ± SEM. Asterisks indicate statistical significance between the control (Group 1) and each infected group as determined by two-way ANOVA and subsequent Dunnett’s multiple comparisons test (* indicates *p* < 0.05, ** indicates *p* < 0.01, *** indicates *p* < 0.001, and **** indicates *p* < 0.0001).

For the reinfection study, the recovered ferrets were allocated into four different groups based on their corresponding NAb titres; group 2 for NAb titre < 20 (*n* = 6), group 3 for NAb titre 20–40 (*n* = 4), group 4 for NAb titre 80 (*n* = 4), and group 5 for NAb titre 160 (*n* = 4). In addition, a naïve seronegative ferret group (*n* = 4) was designated as group 1, the infection control group (Figure 1B). At 4 weeks from the initial SARS-CoV-2 infection, each group of ferrets were infected with 10^5.0^ TCID_50_ of heterologous CBNU-nCoV02 strain (GH clade), which shows 99.9% amino acid identity with NMC-nCoV02 (Table 1). Virus copy numbers were evaluated every other day in nasal washes and rectal swabs of each group of ferrets. The viral copy number in nasal washes of control group 1 peaked at 4 dpi (4.78 ± 0.35 log_10_ copies/mL) and persisted until 8 dpi (1.55 ± 0.18 log_10_ copies/mL). Surprisingly, RT–PCR results revealed that the CBNU-nCoV02 RNA was detected as early as 2 days post-reinfection in both nasal washes and rectal swabs in all experimental groups, although their RNA copy numbers varied depending on the NAb titres (Figure 2A and Table 2). The viral copy number in nasal washes of group 2 peaked at 4 dpi (3.17 ± 0.58 log_10_ copies/mL) and viral RNA was persistently detected until 8 dpi (0.40 ± 0.22 log_10_ copies/mL) before dropping below the level of detection at 10 dpi. Groups 3 and 4 also showed the highest virus RNA copy numbers at 4 dpi with 3.19 and 2.72 log_10_ copies/mL, respectively, suggesting active replication of SARS-CoV-2 in the upper respiratory tract during the reinfection period. However, in group 5, the virus RNA copy number peaked at 2 dpi and gradually decreased until 6 dpi (Figure 2A).

**Table 2.** Viral copy number of reinfected ferrets.

NAb titre groups (*n*)	2dpi	4dpi	6dpi	8dpi	10dpi
	Viral RNA quantitation (log_10_copies/mL)				
*Rectal swab*					
Group1 (*n *= 4)	1.39 ± 0.15	1.56 ± 0.19	0.72 ± 0.27	0.47 ± 0.18	<0.3
Group2 (*n *= 6)	1.30 ± 0.20	1.91 ± 0.48	0.44 ± 0.14	<0.3	<0.3
Group3 (*n *= 4)	1.16 ± 0.32	0.94 ± 0.54	0.43 ± 0.09	<0.3	<0.3
Group4 (*n *= 4)	0.93 ± 0.10	0.92 ± 0.57	0.35 ± 0.02	<0.3	<0.3
Group5 (*n *= 4)	0.76 ± 0.80	0.58 ± 0.48	<0.3	<0.3	<0.3

Notes: SARS-CoV-2 spike RNA gene detection limit and viral titre limit were 0.3 log_10_ copies/mL.

A similar pattern of viral RNA copy numbers was observed in rectal swab specimens of each group as in the nasal wash specimens. Most of the reinfection groups showed the highest virus titres at 2 dpi, which gradually decreased until 6 dpi, with exception of group 2, which showed the highest virus RNA copy number of 1.91 log_10_ copies/mL at 4 dpi (Table 2).

**Figure 3.**
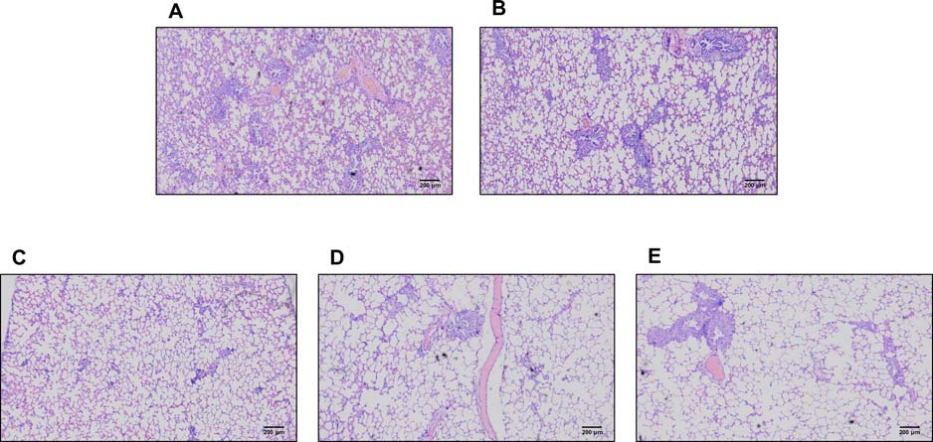
Histopathology of lungs following reinfection. Previously infected ferrets were inoculated with 10^5.0^ TCID_50_ of CBNU-nCoV02 (GH clade) virus. Tissues were harvested on day 6 after inoculation. Group 1 (control group) (A), Group 2 (NAb titre < 20) (B), Group 3 (NAb 20–40) (C), Group 4 (NAb titre 80) (D), and Group 5 (NAb titre 160) (E). Magnification 40X.

To evaluate the infectious virus titre in each specimen, collected nasal washes were inoculated with Vero cells for virus isolation. The CBNU-nCoV02 virus was continuously isolated from group 1 ferrets from 2 dpi (4/4) until 6 dpi (4/4) (Figure 2B). In group 2, infectious virus was isolated at 2 dpi (4/5) and 4 dpi from two out of five reinfected ferrets. However, no virus was isolated from the rest of the groups although qRT-PCR results revealed that the CBNU-nCoV02 RNA was detectable in all groups of ferrets in both the nasal wash and rectal swab specimens (Figure 2B and Table 2). Further, lung histopathology demonstrated the presence of limited inflammatory regions in high NAb titre groups compared with control and low NAb groups (Figure 3). These results indicate that high NAb titre is associated with low infectious virus titre and rapid viral clearance in the ferret reinfection model.

To investigate whether reinfected ferrets could shed infectious virus, naïve sentinel ferrets (*n* = 3) were co-housed with each group at 2 dpi for direct contact transmission and nasal washes were collected daily for virus isolation. The CBNU-nCov02 virus was recovered from all ferrets of group 1 (control) and two ferrets of group 2 (NAb titre < 20) from 2 days post-contact (dpc). However, no virus was detected from the nasal washes of ferrets co-housed with the rest of the groups (Figure 2B). This demonstrates that reinfected groups 3, 4 and 5, all with high NAb titres (20<), did not shed infectious virus at levels high enough for transmission via direct contact to naïve ferrets.

### Changes in NAb responses of reinfected ferrets

**Figure 4.**
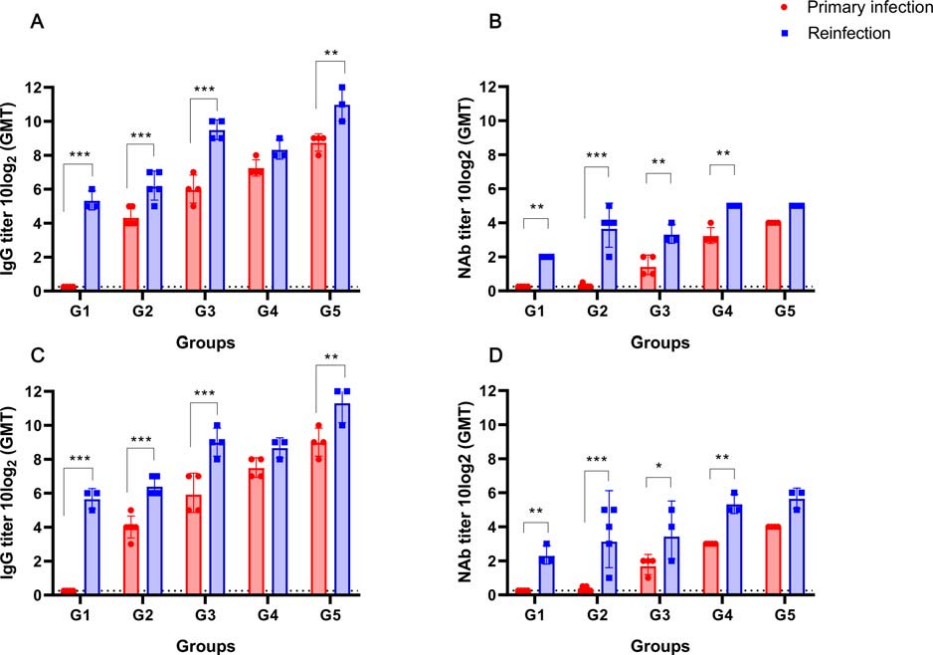
Comparison of total IgG and NAb titres between primary infection and reinfection of ferrets. Immunofluorescence assay (IFA) was performed with sera of SARS-CoV-2 infected ferrets using fluorescein-labeled anti-ferret IgG antibody (A and C). Vero cells were infected with 1 × 10^3^ TCID_50_/mL NMC-2019-nCoV02 (S clade) (A), CBNU-nCoV02 (GH clade) (C) and incubated with serially diluted ferret sera. Fluorescein-labeled anti-ferret IgG was used as the secondary antibody. NAb titre against NMC-2019-nCoV02 (100 TCID_50_) (B) and CBNU-nCoV02 (100 TCID_50_) (D) were measured using Vero cells (B and D). Data are presented as geometric mean ± SD. Asterisks indicate statistical significance compared with primary infection sera by two-way ANOVA Sidak’s multiple comparisons test (* indicates *p* < 0.05, ** indicates *p* < 0.01, and *** indicates *p* < 0.0001).

To evaluate and compare the levels of the serum IgG antibody and the NAb titre between the first infection (NMC-2019-nCoV02) and reinfection (CBNU-nCoV02) groups, serum specimens were collected at the primary infection (0 dpi) and after the reinfection period (14 dpi). Immunofluorescence analysis (IFA) revealed at least a four-fold increase in IgG levels from 0 to 14 dpi against both viruses. Notably, a significant increase in IgG titre was observed in groups 1, 2, 3 (*p* < 0.0001), and 5 (*p* < 0.01) (Figure 4A,C). For the NAb assay, the highest fold changes were observed in group 2 (more than 8 fold (*p* < 0.0001)) followed by group 4 (4 fold (*p* < 0.01)) (Figure 4B,D). Although one or two individual ferrets in groups 2 and 3 demonstrated little or no increase in antibody titres after reinfection, most of the reinfection groups showed significantly enhanced NAb titres against both viruses (Figure 4B,D). It should be noted that there was no significant difference in the degree of antibody response against NMC-2019-nCoV02 (S clade) and CBNU-nCoV02 (GH clade). These results suggest that active reinfection with heterologous SARS-CoV-2 occurs in ferrets regardless of their serum antibody status.

## Discussion

NAbs are considered to be a valuable indicator of protective immunity against reinfection after the clearance of a primary infection [21]. Recently, Okba et al. reported that the serum antibody levels against SARS-CoV-2 were higher in severe COVID-19 patients than in asymptomatic to mild patients [22]. However, to date there is no substantial evidence indicating whether naturally recovered COVID-19 patients are protected against re-exposure to heterologous SARS-CoV-2. If susceptible to reinfection, it is possible recovered patients might act as SARS-CoV-2 reservoirs for continuous spread. To address these globally important details of SARS-CoV-2 reinfection, we adapted the ferret model, which is highly susceptible to SARS-CoV-2 infection and transmission [19,20], and evaluated the potential for reinfection by probing virus titre, shedding period, transmission, and antibody responses following reinfection. These studies revealed that all ferrets reinfected with heterologous SARS-CoV-2 showed detectable virus replication in nasal washes and rectal swabs regardless of their primary antibody titres, suggesting that primary infection-induced high NAb titres may not completely protect the host from reinfection with a heterologous strain. However, it is important to note that ferrets with high NAb titres showed attenuated viral RNA levels in both respiratory and gastrointestinal tracts, which led to rapid viral clearance compared to the control group. In this study, we primarily focused on the NAb-dependent cross-protection against heterologous SARS-CoV-2 infections. While T cell responses were expected to contribute to the attenuation of SARS-CoV-2 replication in infected ferrets along with strong humoral responses, we could not measure T cell responses against SARS-CoV-2 due to the lack of ferret-specific immunological reagents.

In addition, recent studies have reported that macaques reinfected with homologous SARS-CoV-2 showed low or no detectable viral RNA in their upper respiratory tracts [12,23], indicating the induction of protective immunity against homologous SARS-CoV-2 reinfection in rhesus monkeys. Although these results are seemingly in contrast with our study, it should be noted that both rhesus macaque studies used a homologous strain of SARS-CoV-2 for both primary infection and reinfection, while in the current study a heterologous CBNU-nCoV02 strain was used for reinfection of ferrets. As SARS-CoV-2 viruses have already been grouped into six different clusters [24–26], understanding the potential for and consequences of reinfection with SARS-CoV-2 with sequence variations is crucial for understanding the development of protective immunity. Unlike our study, which utilized ferret groups infected with a range of doses of SARS-CoV-2 resulting in varied NAb titres, only rhesus monkeys with high NAb titres were used for the reinfection studies with homologous SARS-CoV-2. Finally, similar to reinfected ferrets (Figure 2), reinfected rhesus monkeys also showed low levels of initial viral replication following reinfection [12,23]. Collectively, these results indicate that NAb titre after the primary infection is a critical determinant in providing protection against reinfection with heterologous SARS-CoV-2 strains.

A major concern with regards to SARS-CoV-2 reinfection is the potential for asymptomatic, reinfected patients to act as transmission reservoirs for virus spread within the local community. In this study, we showed that limited transmission was observed only in the ferrets with NAb titres less than 20, whereas no transmission was seen in ferrets with NAb titres greater than 20. It is noteworthy that while relatively high viral RNA copy numbers were detected in all heterologous SARS-CoV-2 infected ferrets for 4 dpi (Figure 2A), infectious viruses were detected only in group 1 (control) and group 2 (NAb < 20). Moreover, only groups 1 and 2 showed transmission to naïve ferrets. This suggests that the detection of viral RNA in clinical specimens does not always indicate the presence of infectious virus and therefore, the detection of both viral RNA and infectious virus should be considered for the evaluation of virus shedding periods.

In addition to respiratory droplet transmission, we have recently demonstrated the presence of infectious viruses in fecal specimens of infected ferrets and COVID-19 patients [19,27] which showed high RNA copy numbers (more than 1.0 log_10_copies/mL). The viral RNA copy numbers in heterologous SARS-CoV-2 infected groups 2 and 3 showed more than 1.0 log_10_copies/mL in rectal swabs at 2–4 dpi, suggesting the potential for infectious virus shedding through the gastrointestinal tract in ferrets with low NAb. Therefore, a certain level of neutralizing antibodies must be present in recovered COVID-19 patients in order to ensure that they do not become a transmission reservoir for community spread of SARS-CoV-2.

As the COVID-19 pandemic continues to spread worldwide, a majority of people infected with SARS-CoV-2 will recover from the primary infection. In order to hamper the continuous spread of this virus, ideally recovered patients would have sufficient neutralizing antibodies to protect themselves against reinfection with heterologous SARS-CoV-2 strains. Recent studies have reported that asymptomatic COVID-19 patients exhibit lower antibody responses than patients with severe COVID-19. Moreover, there is rapid decline of anti-SARS-CoV-2 antibody responses in asymptomatic COVID-19 patients compared with severe COVID-19 patients [28–30]. Thus, asymptomatic patients may be at high risk of reinfection and subsequent transmission. Our ferret reinfection model with dose-dependent induction of SARS-CoV-2 NAbs provides detailed insight into the possibility of reinfection in humans and emphasizes a close correlation between neutralizing antibody titre and SARS-CoV-2 reinfection. Therefore, further investigation of the correlation between reinfection and neutralizing antibodies is essential to facilitate the development of vaccine platforms and epidemiological protocols against SARS-CoV-2 infection.
